# Elevated Pressure Improves the Extraction and Identification of Proteins Recovered from Formalin-Fixed, Paraffin-Embedded Tissue Surrogates

**DOI:** 10.1371/journal.pone.0014253

**Published:** 2010-12-08

**Authors:** Carol B. Fowler, Ingrid E. Chesnick, Cedric D. Moore, Timothy J. O'Leary, Jeffrey T. Mason

**Affiliations:** 1 Department of Biophysics, Armed Forces Institute of Pathology, Rockville, Maryland, United States of America; 2 Biomedical Laboratory Research and Development Service, Veterans Health Administration, Washington, D.C., United States of America; Indiana University, United States of America

## Abstract

**Background:**

Proteomic studies of formalin-fixed paraffin-embedded (FFPE) tissues are frustrated by the inability to extract proteins from archival tissue in a form suitable for analysis by 2-D gel electrophoresis or mass spectrometry. This inability arises from the difficulty of reversing formaldehyde-induced protein adducts and cross-links within FFPE tissues. We previously reported the use of elevated hydrostatic pressure as a method for efficient protein recovery from a hen egg-white lysozyme tissue surrogate, a model system developed to study formalin fixation and histochemical processing.

**Principal Findings:**

In this study, we demonstrate the utility of elevated hydrostatic pressure as a method for efficient protein recovery from FFPE mouse liver tissue and a complex multi-protein FFPE tissue surrogate comprised of hen egg-white lysozyme, bovine carbonic anhydrase, bovine ribonuclease A, bovine serum albumin, and equine myoglobin (55∶15∶15∶10∶5 wt%). Mass spectrometry of the FFPE tissue surrogates retrieved under elevated pressure showed that both the low and high-abundance proteins were identified with sequence coverage comparable to that of the surrogate mixture prior to formaldehyde treatment. In contrast, non-pressure-extracted tissue surrogate samples yielded few positive and many false peptide identifications. Studies with soluble formalin-treated bovine ribonuclease A demonstrated that pressure modestly inhibited the rate of reversal (hydrolysis) of formaldehyde-induced protein cross-links. Dynamic light scattering studies suggest that elevated hydrostatic pressure and heat facilitate the recovery of proteins free of formaldehyde adducts and cross-links by promoting protein unfolding and hydration with a concomitant reduction in the average size of the protein aggregates.

**Conclusions:**

These studies demonstrate that elevated hydrostatic pressure treatment is a promising approach for improving the recovery of proteins from FFPE tissues in a form suitable for proteomic analysis.

## Introduction

Proteomic methods are widely employed for a variety of applications, including disease biomarker discovery, [1]–[4], elucidation of physiological processes [5], and localization of post-translational modifications [6], [7]. For example, malignant cells yield unique “protein profiles” when total protein extracts from such cells are analyzed by 2-D gel electrophoresis or mass spectrometry (MS) methods. Such proteomic studies have the potential to provide an important complement to the analysis of DNA and mRNA extracts from these tissues [8]. Large cohorts of fresh or frozen tissue are often difficult to obtain, and when used for proteomic analyses, the results generally cannot immediately be related to the clinical course of diseases. If the millions of available fixed and embedded archival tissues could be used for standard proteomic methods such as MS, these powerful techniques could qualitatively and quantitatively analyze large numbers of tissues for which the clinical course of disease has been established. However, the extraction of proteins from archival formalin fixed, paraffin-embedded (FFPE) tissues for proteomic analysis has been hampered by the deleterious effects of formaldehyde-induced protein adducts and cross-links that are formed during tissue fixation and subsequent histological processing.

Three types of formaldehyde-induced chemical modifications have been identified in proteins and model peptides: (a) methylol (hydroxymethyl) adducts, (b) Schiff's bases, and (c) stable methylene bridges [9], [10]. Formaldehyde can react with lysine, cysteine, arginine, tryptophan, histidine, and the *N*-terminal amine to form methylol adducts. The methylol adduct can subsequently undergo a dehydration reaction to form a Schiff's base, which is seen most frequently in lysine and tryptophan residues. Additionally, the protein *N*-terminal amine can be converted to a stable 4-imidazolidione adduct [9] and a Mannich reaction can occur between adducted tyrosine and arginine residues in close spatial proximity [11]. Intramolecular protein cross-links (methylene bridges) have been reported in both model peptides [10] and whole proteins, such as insulin [9].

Several proteomic studies using archival FFPE tissues have been reported in recent years. Some involve the analysis of a very small number of cells prepared by laser-capture microdissection from FFPE tissue sections [12]–[14]. The majority of the proteomic studies on FFPE tissues employ tissue extraction methods that are derived from heat-induced antigen retrieval (AR) methods originally developed for immunohistochemistry. A number of recent studies report improved identification of proteins from FFPE tissue using these AR-based methods, which employ combinations of heat and recovery buffers containing Tris-HCl [15], detergents[16]–[18] and reducing agents such as DTT [19], [20]. However, these studies do not systematically address the issue of protein quality and mechanism of protein recovery. A comparison of published extraction methods established the importance of heat, detergent, and a protein denaturant for efficient protein extraction from FFPE tissues [21], though in this study detergent alone was as effective as buffers containing reducing agents.

Our studies with model FFPE tissue surrogates [21], and formaldehyde-fixed proteins [22] showed that these AR-based methods did not completely reverse formaldehyde-induced protein cross-links. When tissue surrogates composed of hen egg-white lysozyme were heated at 80°C for 2 h at ambient pressure, the protein extraction efficiency was relatively low, with 60% of the total protein extracted at pH 4, 51% at pH 6, and 49% at pH 9. In addition, the lysozyme remained highly cross-linked [21]. In contrast, when the lysozyme tissue surrogate was heated at 80°C for 2 h at elevated pressures (43,500 psi), 100% of the protein was recovered in the soluble phase regardless of pH, and complete reversal of the formaldehyde-induced protein adducts and cross-links was observed at pH 4 [23]. In this study, we report the improved extraction of proteins from FFPE mouse liver and a multi-protein FFPE tissue surrogate consisting of five proteins using a combination of heat and elevated hydrostatic pressure. Protein identity, sequence coverage, and false identification rates were evaluated by liquid chromatography-MS (LC/MS). In addition, studies were performed to investigate the effect of pressure on the rate of reversal of formaldehyde-induced protein adducts and cross-links and on the size of the protein aggregates recovered from the tissue surrogates. The results of these studies provide insight into the mechanism of pressure-enhanced protein recovery from FFPE tissues.

## Materials and Methods

Chicken egg white lysozyme, bovine carbonic anhydrase, bovine ribonuclease A, bovine serum albumin (BSA), equine myoglobin, sodium dodecyl sulfate (SDS), dithiothreitol (DTT), iodoacetamide (IAA), formic acid, phosphate buffer, and Tris-HCl buffer were purchased from Sigma (St. Louis, MO, USA). High-pressure liquid chromatography (HPLC) grade water, aqueous 37% formaldehyde, and xylene were purchased from Thermo Fisher Scientific (Pittsburgh, PA, USA). HPLC grade acetonitrile was purchased from Honeywell Burdick and Jackson (Muskegon MI, USA). Sequencing grade modified trypsin was purchased from Promega (Madison, WI, USA). Absolute ethanol was purchased from Pharmco-AAPER (Brookfield, IL, USA), and Paraplast tissue embedding medium was purchased from Oxford Labware (St. Louis, MO, USA).

### Formation of FFPE Tissue Surrogates

The FFPE tissue surrogates were prepared as described previously [21], [23]. Briefly, aliquots of a 150 mg/mL solution of lysozyme or a 150 mg/mL solution (total protein) consisting of lysozyme, carbonic anhydrase, ribonuclease A, BSA, and myoglobin (55∶15∶15∶10∶5 w/w) in deionized water were mixed with an equal volume of 20% phosphate-buffered formalin. An opaque gel formed within 2 min, and the tissue surrogate was allowed to sit at room temperature in the presence of formaldehyde for at least 24 h to mimic typical tissue fixation methods. Dehydration and paraffin-embedding were conducted according to standard histological protocols [24]. The tissue surrogate was washed for 10 min with distilled water and then dehydrated through a series of graded alcohols: 70% ethanol for 30 min, 85% ethanol for 30 min, 100% ethanol for 30 min, and a final 100% ethanol dehydration overnight. The tissue surrogate was incubated through two changes of xylene, 30 min each, and placed in 65°C liquid paraffin for 6 hr before embedding.

### Preparation of FFPE Tissue

The liver from a female BALB/c mouse was given as a gift under the secondary use provision by the Department of Veterinary Pathology, Armed Forces Institute of Pathology. The liver was bifurcated with a sterile surgical scalpel and one half was immediately snap-frozen in Tissue-Tek O.C.T. compound (Sakura Finetek). The other half was fixed for 48 h at 4°C in 10% buffered formalin. The formalin fixed tissue was washed for 30 min with distilled water and then dehydrated through a series of graded alcohols and xylenes for 1 h each: (70%, 85%, 100%, and100%) ethanol, and two changes of xylene. The tissue was incubated overnight at 65°C in Paraplast Plus paraffin (Thermo Fisher Scientific) before embedding. The FFPE liver was stored for approximately 11 months prior to sectioning and protein recovery.

### Deparaffinization and Recovery of Multi-protein Tissue Surrogates and FFPE Mouse Liver

10 µm sections of the FFPE tissue surrogates and FFPE liver were deparaffinized by incubating the sections through two changes of xylene for 10 min each. The sections were rehydrated through a series of graded alcohols for 10 min each: 2 changes of 100% ethanol, 85% ethanol, and 70% ethanol, and then incubated in distilled water for a minimum of 30 min.

For routine protein recovery, 6–8 of the rehydrated FFPE liver sections and tissue surrogate sections were resuspended in 6 mL of 50 mM Tris-HCl at pH 4 or 8, with 2% (w/v) SDS. The samples were homogenized with a disposable pellet pestle (Kontes Scientific, Vineland, NJ, USA), followed by two 10 s cycles of sonication on ice using a Sonic Dismembrator, model 550, fitted with a 0.125 inch tapered microtip (Thermo Fisher Scientific). The homogenized FFPE samples were split in half and incubated at 100°C for 30 min followed by 80°C for 2 h at either atmospheric pressure (14.7 psi) or 40,000 psi as previously described [23]. Equivalent sections fresh-frozen mouse liver tissue were homogenized in the Tris-HCl/SDS extraction buffer supplemented with 15 µl/ml of protease inhibitor cocktail (Sigma p8340) and heated at 95°C for three minutes. Briefly, high-pressure experiments were conducted with a 3 ml capacity model MS-1 stainless steel reaction vessel coupled to a manually operated model HiP high pressure hydrostatic generator (High Pressure Equipment Company, Erie, PA, USA). The sample incubation temperature was regulated with a Eurotherm 2132 temperature controller (Leesburg, VA, USA) connected to an aluminum heating collar surrounding the reaction vessel. An inline Gilson model 303 HPLC pump (Middleton, WI, USA) supplied the buffer to be pressurized.

### Effect of Pressure on Aggregate Size

1.5 mg aliquots of lysozyme tissue surrogates were cleared of paraffin and homogenized as described in the previous section. The lysozyme tissue surrogates were heated at 100°C for 2 h in 50 mM Tris-HCl, pH 4, with 2% SDS and 0.2 M glycine. To determine the effect of pressure and heat treatment on protein aggregate size, the tissue surrogate suspensions were heated at 14.7, 2,500, 5,000, 10,000, 15,000, 20,000, 30,000, 40,000, or 50,000 psi. The extracted lysozyme surrogates were cleared by centrifugation. Triplicate samples processed at each pressure were diluted 1∶10 in PBS, pH 7.4, and the average particle size of the recovered lysozyme protein aggregates were measured by dynamic light scattering using a NICOMP model 370 particle sizer (Particle Sizing Systems, Santa Barbara, CA, USA).

### Pressure Dependence of Formaldehyde Adduct Reversal

A 2 mg/mL solution RNase A in phosphate-buffered saline, pH 7.4 (PBS), was treated with an equal volume of 20% formalin in PBS for 1 hour. The formalin-treated, dilute solution remained in solution, unlike the more concentrated tissue surrogate solutions. The excess formaldehyde was removed by dialysis against one change of PBS, pH 7.4, and three changes of Tris acetate/EDTA (TAE) buffer, pH 4 (40 mM Tris, 1 mM EDTA) in Slide-A-Lyzer dialysis cassettes with a molecular weight cut-off of 3.5 kDa (Pierce, Rockford, IL, USA), as previously described [25], [26]. The fixed RNase A solutions were incubated under pressures ranging from 14.7 to 40,000 psi for 3.5 h at either 55°C or 65°C in the model MS-1 reaction vessel. The samples were also incubated at the above range of pressures for 3.5 h at either room temperature or 45°C using a model NEP 2320 Barocycler (Pressure BioSciences, Inc., South Easton, MA, USA).

### Electrophoresis and Analysis of Protein Composition

The protein concentration of the solubilized tissue surrogates, FFPE mouse liver extracts and RNase A solutions were determined spectrophotometrically using a Nanodrop 1000 (Thermo Scientific, Waltham, MA, USA). Pre-cast gels, buffers, molecular weight standards, Coomassie brilliant blue stain and the SilverQuest staining kit were purchased from Life Technologies, Carlsbad, CA, USA. Each sample was analyzed by SDS-polyacrylamide gel electrophoresis (PAGE) using 5–10 µg of dithiothreitol-treated samples in the presence of 0.1% (w/v) SDS. SDS-PAGE was performed on precast NuPAGE Bis-Tris 4–12% gradient polyacrylamide gels using 2-(*N*-morpholino) ethanesulfonic acid-SDS running buffer at pH 7.3, and the gels were stained according to manufacturer's instructions. Gel images were documented using an Epson flat-bed scanner in transparency mode (Long Beach, CA, USA) and annotated in Adobe Photoshop, version 7.1. The composition of the lysozyme and RNase A samples was analyzed by measuring the intensity of the protein monomer and oligomer bands using Un-Scan-it Gel 6.1 analysis software (Silk Scientific Corp., Orem, UT, USA).

### Mass Spectrometry

Multi-protein FFPE tissue surrogate samples (15 µg each) were washed three times with 50 mM NH_4_HCO_3_, pH 7.9, using an Amicon Ultra 3K centrifugal filter (Millipore, Billerica, MA, USA). The excess SDS was removed using an SDS-out detergent precipitation kit (Pierce), and the recovered tissue surrogates were washed against 50 mM NH_4_HCO_3_ an additional 7 times. Acetonitrile was added to a final concentration of 20%, and the samples were denatured at 50°C for 1 h in the presence of 20 mM DTT, then alkylated with 10 mM IAA for 1 h at room temperature in the dark [27]. A solution of the surrogate proteins prior to treatment with formaldehyde (native, unfixed mixture) was also analyzed. Sequencing-grade modified trypsin was added to each vial to give a final concentration of 0.75 µg/mL, and the samples were digested overnight at 37°C. Recovered FFPE tissue surrogate samples were analyzed by reversed-phase liquid chromatography (RPLC) coupled directly in-line with an Agilent 6340 ion trap mass spectrometer (Palo Alto, CA, USA). Microflow RPLC was conducted with an Agilent 1100 LC system using a 0.3 mm (inner diameter) ×15 cm long Zorbax 300 Stable Bond column packed with 3.5 µm, 300 Å pore-size C8 media (Agilent). A binary gradient consisting of 0.1% formic acid in water (A) and 0.1% formic acid in acetonitrile (B) was used as the mobile phase. After injecting 8 µl (4.5 µg) of sample, the column was washed for 10 min (at 10 µl/min) with 2% B, and the peptides were then eluted (at 10 µl/min) using the following gradient: 2–70% B over 136 min, 70–95% B over 1 min, and 95% B for 15 min. The column was re-equilibrated with 2% B for 30 min prior to subsequent sample loading. The mass spectrometer was operated in a data-dependent mode where the three most intense ions detected in each MS scan were selected for tandem MS (MS/MS) in the linear ion trap. The drying gas temperature was 300°C, and normalized collision energy of 1.3 V was employed for collision-induced dissociation along with a dynamic exclusion of 30 s to reduce redundant peptide selection.

Raw MS/MS data were analyzed using the Spectrum Mill Proteomics Work Bench (Agilent) using a UniProtKB/Swiss-Prot combined database containing 517,802 protein sequences (www.expasy.org). Precursor ion tolerance was set to 2.5 Da and fragment ion tolerance was set to 0.75 Da. Only peptides possessing tryptic termini and exhibiting a score of ≥10.5, and a scored peak intensity of ≥70%, were considered legitimate identifications. The peptide searches were conducted allowing for up to two internal missed tryptic cleavage sites.

## Results

### Recovery and Identification of Proteins in a Multi-Protein FFPE Tissue Surrogate

**Figure 1:**
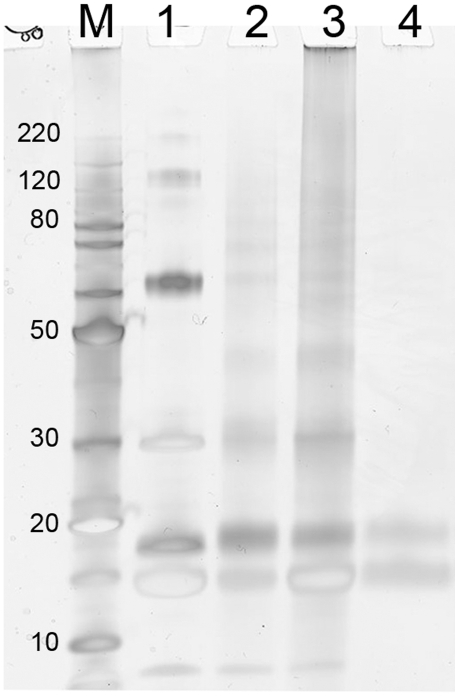
Elevated pressure improves protein extraction from model FFPE tissue surrogates. FFPE tissue surrogates were heated in 50 mM Tris, pH 8+2% SDS at either elevated pressure (40,000 psi) or atmospheric pressure. The electrophoretic mobility of the tissue surrogate extracts were compared to the native, unfixed tissue surrogate mixture by 1D-PAGE. Lane M: molecular weight marker; lane 1: native, unfixed tissue surrogate mixture; lane 2: FFPE tissue surrogate with 2.5% myoglobin after retrieval at 40,000 psi; lane 3: FFPE tissue surrogate with 5% myoglobin after retrieval at 40,000 psi,; lane 4: FFPE tissue surrogate with 5% myoglobin after retrieval at atmospheric pressure (14.7 psi).

When a multi-protein FFPE tissue surrogate consisting of lysozyme, carbonic anhydrase, ribonuclease A, BSA, and myoglobin (55∶15∶15∶10∶5 w/w) was extracted under elevated pressure, ∼96% of the protein was solubilized at pH 4 or 8. This was approximately a 4-fold increase over the same tissue surrogate extracted at atmospheric pressure (Table 1). Additionally, when the pressure-retrieved tissue surrogate mixture was separated by SDS-PAGE (Figure 1, lane 3), there were a number of well-resolved higher and lower molecular weight bands corresponding to those seen in the corresponding native, unfixed protein mixture (Figure 1, lane 1). However, the tissue surrogate extracted at atmospheric pressure (Figure 1, lane 4) appeared to mainly contain bands corresponding to RNase A and lysozyme (14–15 kDa bands) and a band at approximately 20 kDa band. A multi-protein tissue surrogate with 2.5% myoglobin was also retrieved under elevated pressure at pH 8 to show that minor protein components (≤2.5% w/w) were detectable (Figure 1, lane 2).

**Table 1:** Effect of pressure on the recovery of total protein from FFPE tissue surrogates.

Buffer	Pressure (psi)	% Protein recovered
50 mM Tris, pH 4+2% SDS	14.7*	26%
50 mM Tris, pH 4+2% SDS	40,000	96%
50 mM Tris, pH 8+2% SDS	14.7	22%
50 mM Tris, pH 8+2% SDS	40,000	96%

Multi-protein FFPE tissue surrogate samples were incubated at 100°C for 30 min followed by 80°C for 2 h at the indicated pressure. Total protein in the supernatants was assessed spectrophotomerically following recovery.

*Atmospheric pressure.

### Mass Spectrometry

The total protein extracts recovered from the multi-protein FFPE tissue surrogates retrieved at 40,000 psi and at atmospheric pressure were digested with trypsin, and 4.5 µg of each sample was analyzed by LC/MS. A widely used extraction protocol, heating in Tris-HCl buffer with 2% (w/v) SDS at atmospheric pressure [16], [28], [29], resulted in poor protein solubilization and few protein identifications (Table 2). For the samples extracted at pH 4, only lysozyme and RNase A were identified, and none of the component proteins were correctly identified by MS/MS for the surrogate extracted at pH 8. The use of elevated hydrostatic pressure to supplement the extraction protocol improved protein identification significantly. For the samples extracted at 40,000 psi in 50 mM Tris-HCl, 2% (w/v) SDS, pH 8, a total of 37 unique peptides were identified, and each of the five component proteins were identified by 2 or more tryptic peptides (Table 2). Similar results were seen for the tissue surrogate extracted at pH 4 and 40,000 psi. The constituent proteins were identified with 28% to 69% sequence coverage. These results were comparable to those obtained with the native, unfixed protein mixture (Table 2). The list of peptides identified by LC/MS/MS is included as a supporting information file (Supporting Data S1).

**Table 2:** LC/MS analysis for a 5-protein FFPE tissue surrogate extracted under atmospheric or elevated hydrostatic pressure.

Condition	Lysozyme		Carbonic Anhydrase		RNAse A		BSA		Myoglobin	
	Peptide hits *	% SequenceCoverage**	Peptide hits	% SequenceCoverage	Peptide hits	% SequenceCoverage	Peptide hits	% SequenceCoverage	Peptide hits	% SequenceCoverage
Native protein mixture	67/10	66%	25/10	56%	10/6	63%	34/23	54%	6/5	38%
FFPE; pH 4, 40 Kpsi	26/8	69%	9/7	36%	12/5	59%	21/12	26%	3/3	28%
FFPE; pH 4, 14.7 psi	4/1	15%	n.d		1/1	7%	n.d		n.d	
FFPE; pH 8, 40 Kpsi	75/7	57%	12/7	30%	11/5	71%	23/15	29%	3/2	16%
FFPE; pH 8, 14.7 psi	n.d		n.d		n.d		n.d		n.d	

Multi-protein FFPE tissue surrogates were extracted at 100°C for 30 min followed by 80°C for 2 h at either 40,000 psi or atmospheric pressure (14.7 psi) in 50 mM Tris-HCL, 2% (w/v) SDS buffer, pH 4 or 8. The extracts were washed extensively, and digested overnight with trypsin at 37°C in 50 mM NH_4_HCO_3_, pH 7.9 with 20% acetonitrile (v/v).

*Peptide hits: total spectra/number of unique peptides.

**%Sequence coverage: percent of theoretical tryptic peptides identified by LC/MS/MS. n.d. – none detected.

**Figure 2:**
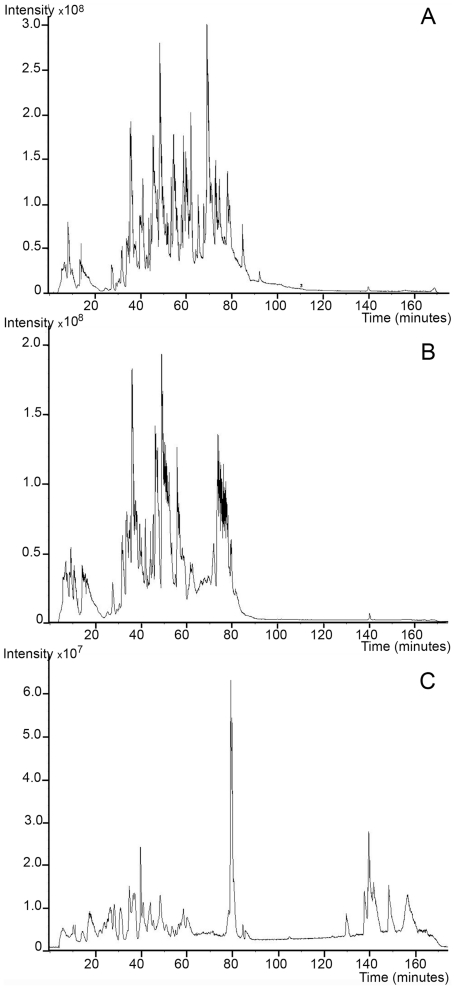
Quality comparison of MS profiles of native protein mixture and tissue surrogate extracts. FFPE tissue surrogates were heated in 50 mM Tris, pH 8+2% SDS at either elevated pressure (40,000 psi) or atmospheric pressure. The extracts were analyzed by LC/MS and the MS traces of each tissue surrogate extract was compared to the native, unfixed protein mixture. A) native, unfixed tissue surrogate mixture; B) FFPE tissue surrogate retrieved at 40,000 psi; C) FFPE tissue surrogate retrieved at atmospheric pressure (14.7 psi).

Analysis of the raw MS data also revealed several differences in the quality of the tissue surrogate extracts. Since the tissue surrogates consisted of a defined set of proteins, it was possible to calculate the average false protein identification rate (number of non bovine, equine or Gallus proteins identified by MS/MS) for each sample, as shown in Table 3. The false identification rates for the pressure extracted multi-protein surrogate samples were 5.7% (pH 8) and 7.8% (pH 4), which was comparable to the native, unfixed protein mixture, with a false identification percentage of 3.3%. The false identification rate for the non-pressure extracted tissue surrogates was 42% (pH 4) and 100% (pH 8). There were also fewer total peptides identified in the non-pressure treated samples, approximately 10% the number identified for the pressure treated and native, unfixed protein samples (data not shown). A comparison of the raw MS spectra of the native protein mixture, pressure-extracted, and non-pressure extracted multi-protein surrogate samples also showed differences in protein quality (Figure 2). The MS profile of the unfixed protein mixture (panel A) exhibited a number of well defined peaks eluting between 10 and 40% acetonitrile (20 to 80 minutes), which is typical of a tryptic peptide digest. The profile for the tissue surrogate extracted under elevated pressure (panel B) also shows a number of peaks eluting between 20–80 minutes. The non-pressure treated surrogate mixture's spectrum (panel C) was reduced in intensity and had several later-eluting peaks, suggesting that a significant proportion of the material was undigested or remained cross-linked.

**Table 3:** Analysis of LC/MS/MS data. Percent of false protein identifications for each sample.

Sample type	Buffer pH	Extraction Pressure	% False protein IDs*
Native, unfixed protein mixture	N/A	N/A	3.3±0.6
Tissue surrogate	4	14.7 psi **	42±4.0
Tissue surrogate	4	40,000 psi	7.8±1.5
Tissue surrogate	8	14.7 psi	100
Tissue surrogate	8	40,000 psi	5.7±1.1

*Determined as percentage of proteins incorrectly identified for spectra with scores ≥10.5, for 2 technical replicates.

**Atmospheric pressure. N/A =  not applicable.

### Recovery of proteins from FFPE mouse liver

**Figure 3:**
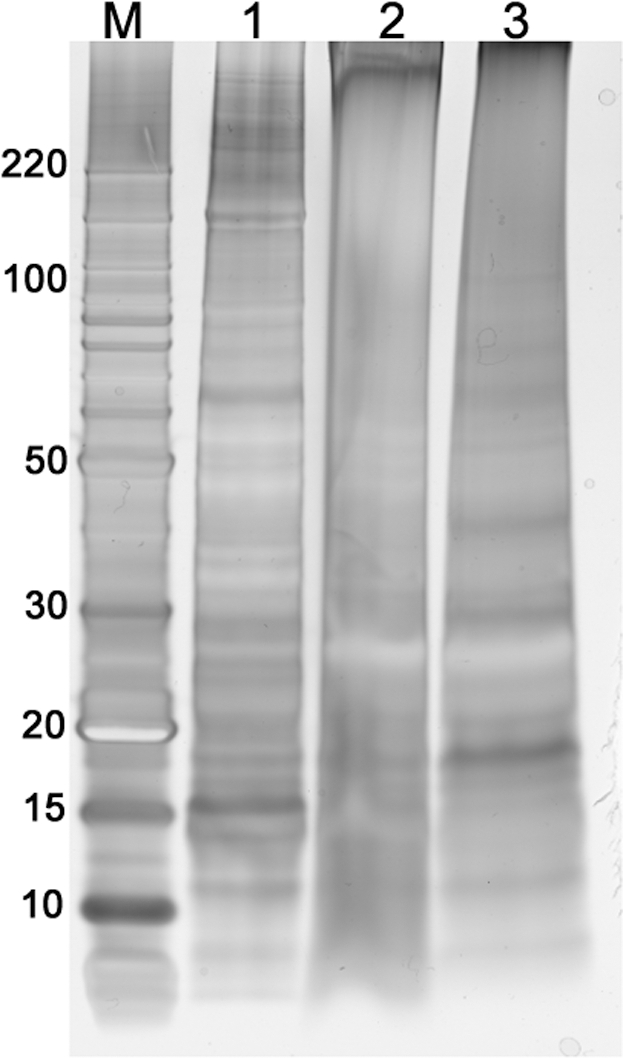
Elevated pressure improves protein extraction from FFPE mouse tissue. Eleven month old FFPE mouse liver tissue was heated in 50 mM Tris, pH 8+2% SDS at either elevated pressure (40,000 psi) or atmospheric pressure (14.7 psi). The electrophoretic mobility of 15 µg of Fresh and FFPE tissue extracts were compared by 1D-PAGE. Lane M: molecular weight marker; lane 1: fresh tissue extract; lane 2: FFPE tissue after retrieval at atmospheric pressure (14.7 psi); lane 3: FFPE tissue surrogate with after retrieval at 40,000 psi.

When FFPE mouse liver tissue was extracted was extracted with heat and under elevated pressure, approximately 77% of the protein was solubilized relative to fresh tissue. Only 17% of total protein was recovered in split samples of FFPE mouse liver tissue heated at atmospheric pressure. When the pressure-retrieved FFPE liver was separated by SDS-PAGE (Figure 3, lane 3), there were a number of well-resolved higher and lower molecular weight bands comparable to those seen in fresh liver extract (Figure 3, lane 1). However, there were fewer well-resolved protein bands seen in equivalent amounts of FFPE tissue heated at atmospheric pressure (Figure 3, lane 2), with most visible protein bands migrating at 10–40 KDa.

### Effect of Pressure on Formaldehyde-Cross-Link Reversal and Protein Aggregate Size

**Figure 4:**
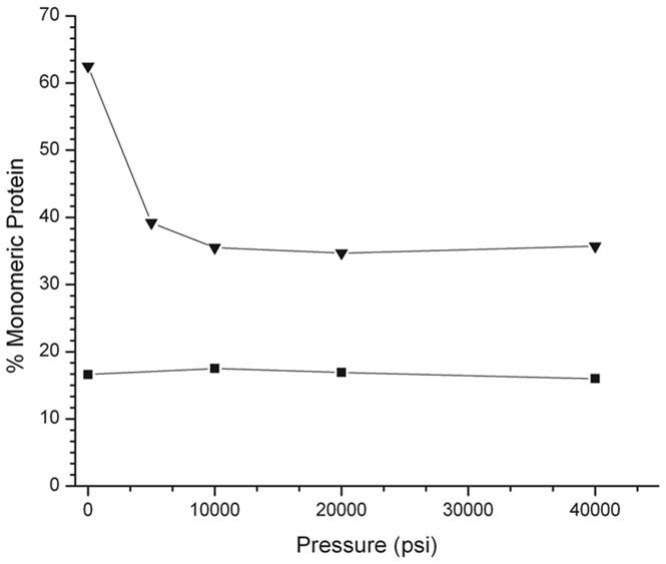
Effect of elevated pressure on the rate of cross-link reversal. Percent monomeric protein recovered. 1 mg/ml solutions of RNase A was incubated in 10% phosphate buffered formalin for one hour, and the excess formaldehyde solution was exchanged for 1× TAE buffer, pH 4. The aqueous fixed RNase A solution consisted of 18 percent monomeric and 82 percent multimeric protein by 1-D SDS-PAGE. Aliquots of the formalin fixed solution were incubated at 14.7–40,000 psi for 3.5 hours at either 55°C (squares) or 65°C (triangles). The heat-treaed samples were separated by SDS-PAGE and the gel bands were integrated to determine the percentage of monomeric protein at each pressure.

We investigated the effect of elevated pressure on the rate of reversal of formaldehyde-induced protein cross-links by incubating solutions of formaldehyde fixed-RNase A at either 55 or 65°C in TAE buffer, pH 4, for 3.5 h under pressures ranging from 14.7–40,000 psi. Solutions of formaldehyde-treated RNase A were used for these experiments to avoid any complications associated with using insoluble FFPE tissue surrogates. At 55°C, the ratio of monomeric/oligomeric protein was independent of pressure, with approximately 82% of the RNase migrating as cross-linked oligomers and 18% as monomeric protein as measured by integration of the SDS-PAGE gel bands (Figure 4). This ratio was consistent from room-temperature to 55°C (data not shown). When the fixed protein solution was incubated at ambient pressure and 65°C, the majority of the cross-links were reversed, with 62% of the protein migrating as monomer and 36% migrating as protein dimer. This was consistent with our previous studies of formaldehyde-fixed RNase A[22], [25], [26]. However, when the fixed RNase A was incubated at 65°C and 5,000 to 40,000 psi, there was a decrease in the rate of cross-link reversal, with only 36–40% of the total protein migrating as the monomeric species (Figure 4).

**Figure 5:**
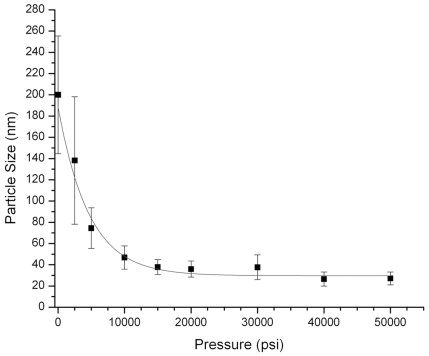
Effect of elevated pressure on aggregate size. Lysozyme tissue surrogates were incubated in 50 mM Tris-HCl buffer, pH 4 with 2% SDS and 0.1 M glycine at100°C for 2 h at pressures ranging from atmospheric pressure (14.7 psi) to50,000 psi. The average particle size of the solubilized protein was measured by dynamic light scattering to determine the degree of protein aggregation.

**Figure 6:**
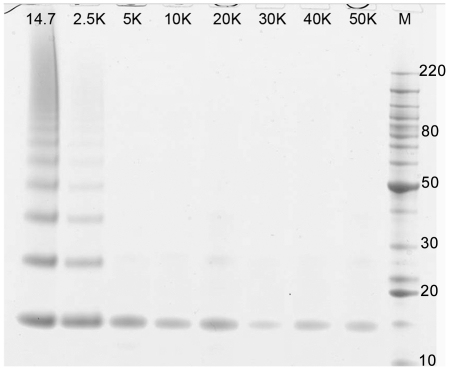
Effect of elevated pressure and temperature on the reversal of intermolecular protein cross-links in lysozyme FFPE tissue surrogates. The tissue surrogates from figure 5 were incubated in 50 mM Tris-HCl buffer, pH 4 with 2% SDS and 0.1 M glycine at100°C for 2 h at pressures ranging from atmospheric pressure (14.7 psi) to50,000 psi buffer at100°C for 2 h at pressures ranging from atmospheric pressure (14.7 psi) to 50,000 psi. The heated treated tissue surrogate samples were separated by 1-D SDS-PAGE. Pressure values are shown at the top of each gel lane.

Lysozyme tissue surrogates were homogenized in 50 mM Tris, pH 4, 2% (w/v) SDS, 0.2 M glycine and incubated at 100°C for 2 h at atmospheric pressure or under pressures ranging from 2,500 to 50,000 psi. After processing, the extracted tissue surrogates were diluted 1∶10 in PBS and the average particle size was determined by dynamic light scattering. The average size of the protein aggregates extracted from surrogates at atmospheric pressure was 200±55 nm. There was a marked decrease in particle size for surrogates extracted over the pressure range of 2,500 (140±57 nm) to 5,000 psi (75±20 nm). For samples extracted at 10,000 psi and above, the particle size was 40–50 nm, as shown in Figure 5. The corresponding SDS-PAGE gel profiles of the extracted proteins (Figure 6) showed that the degree of cross-link reversal at 100°C was directly proportional to increasing pressure. The surrogate extracted at atmospheric pressure remained highly cross-linked. However, the inter-molecular cross-links were almost completely reversed at pressures above 10,000 psi.

## Discussion

We previously showed improved extraction and analysis of proteins from FFPE tissue surrogates by the addition of elevated hydrostatic pressure to conventional heat-induced antigen retrieval extraction protocols [23]. To better mimic the complex mixture of proteins in tissue, we constructed an FFPE tissue surrogate consisting of five proteins with varying abundances, molecular weights (MW), isoelectric points (pI), and secondary structures. RNase A (15% w/w; MW 13.7 kDa) and lysozyme (55% w/w; MW 14 kDa) are both members of the α+β structural class [30], [31], with pIs of 9.7 and 11.2, respectively. Because lysozyme and RNase A contain high percentages of lysine and arginine, they are known to form intermolecular and intra-molecular cross-links in the presence of formaldehyde [21], [25], [26]. Myoglobin (5% w/w; MW 17 kDa; pI of 7.0), is an all-alpha helical protein [32], while carbonic anhydrase (15% w/w; MW 29 kDa; pI of 6.3) has an all-beta secondary structure [33]. BSA (10% w/w; MW 66 kDa; pI 4.7) is an alpha-helical protein with 17 disulfide bonds [34], [35].

The addition of high hydrostatic pressure (40,000 psi) to augment heat treatment (100°C for 30 min, followed by 80°C for 2 h) dramatically improved protein extraction efficiency from multi-protein FFPE tissue surrogates (from ∼25% to 96%). By SDS-PAGE, the high-pressure extracted tissue surrogate sample consisted of a number of well-resolved bands with the same mobility as the unfixed component proteins (Figure 1). In contrast, only lower molecular species were extracted at low (atmospheric) pressure (Figure 1, lane 4).

To test the applicability of our method for archival tissue, we extracted 11-month old FFPE mouse liver using the pressure-assisted protocol developed for our model systems. The results were consistent with those seen for the multi-protein tissue surrogate, with an observed 4.5-fold increase in protein extraction efficiency for tissue extracted with heat and elevated pressure over tissue extracted with heat alone. SDS-PAGE of equal amounts of total protein shows that the liver tissue extracted at 40,000 psi consists of a range of well-defined high and low molecular weight bands (Figure 3, lane 3). For both the tissue surrogate and FFPE liver heated at atmospheric pressure, there appeared to be a bias for the extraction of lower molecular weight species. This phenomenon is most likely due to the lower extraction efficiency seen with the low-pressure extracted samples and an incomplete solubilization of high molecular weight protein complexes and oligomers.

Because the multi-protein tissue surrogate has a defined protein composition, we employed this system for our quality evaluation by LC/MS. We found that the addition of elevated pressure to a well established heat-induced protein extraction protocol [16] improved the proteomic analysis of the FFPE tissue surrogate. For example, the LC/MS trace of the tryptic digests of the surrogates extracted at atmospheric pressure with heat at pH 4 or 8 showed a number of broad, late eluting peaks, suggesting that the material was either poorly digested, or remained cross-linked (Figure 2, panel C). There were only a total of 5 correctly identified spectra, representing 2 unique peptides (false ID rate of 42%), for the surrogate extracted at pH 4 and ambient pressure, and no correctly identified tryptic peptides for the surrogate extracted at pH 8 and ambient pressure (Tables 2 and 3). The surrogates extracted with heat and elevated pressure compared favorably with the corresponding native, unfixed protein mixture. The tryptic digest for both the unfixed protein mixture and pressure-extracted samples eluted between 10 and 40% acetonitrile with no late-eluting peaks. The sequence coverage map (percent of theoretical tryptic peptides identified for each component protein) suggested that essentially unmodified proteins were retrieved from the pressure-extracted FFPE tissue surrogates. BSA, which is known to form cross-links with lysozyme in solution [36], was identified with 29% (pH 8) and 26% (pH 4) sequence coverage when extracted from the multi-protein FFPE tissue surrogate at 40,000 psi (Table 2). RNase A and lysozyme, which have a high number of formaldehyde –reactive residues, were identified with sequence coverages comparable to the native protein mixture (59 and 69% sequence coverage at pH 4 at 40,000 psi, respectively). Myoglobin, which was included as a low-abundance component, was identified by 2 or more fully tryptic peptides in the pressure-extracted multi-protein FFPE tissue surrogates.

There is a sound thermodynamic basis for hypothesizing that increased hydrostatic pressure, along with heat, will facilitate the extraction of proteins from FFPE tissues. Under elevated pressure, cavities in proteins become filled with water molecules, which leads to the hydration of the protein interior [37], [38]. Hydration of the buried hydrophobic residues induces protein unfolding because unfolding reduces the protein's molar volume [39].

We next investigated the mechanism of pressure-assisted protein recovery using two model systems: dilute, aqueous solutions of RNase A [25], [26], and solid-single protein tissue surrogates [21], [23]. By previous observation, augmenting heat treatment with elevated pressure appeared to improve protein-formaldehyde cross-link reversal as well as total protein solubilization [23]. To determine if pressure was accelerating formaldehyde adduct reversal, soluble solutions of formalin-fixed RNase A were heated at 55°C or 65°C for 3.5 hours at 14.7–40,000 psi so that the rate of intermolecular cross-link reversal could be studied independent of protein solubilization, which would not be possible using tissue surrogates. At 55°C, the percent of monomeric protein was constant, with approximately 82% of the RNase migrating as cross-linked oligomers and 18% of protein migrating as monomeric protein as measured by SDS-PAGE. When the fixed RNase A solutions were incubated at 1 atmosphere and 65°C, the majority of the intermolecular cross-links were reversed, with 62% of protein migrating as monomeric protein by SDS-PAGE. However, at pressures between 5,000–40,000 psi, the amount of monomeric protein decreased to 40–36% of the total protein. These results suggest that the application of elevated pressure does not enhance protein recovery from FFPE tissue by accelerating the rate of formaldehyde adduct reversal. Instead, the reaction rate was modestly decreased by pressure, which may be explained by other studies in which elevated pressure has been shown to protect proteins from thermal denaturation [40] and to inhibit other chemical reactions, such as the Maillard reaction between glucose and lysine [41].

To investigate the effect of pressure on protein solubilization, we used a lysozyme tissue surrogate to examine the effects of elevated pressure on average protein aggregate size. Because lysozyme has a higher than average percentage of formaldehyde-reactive residues than FFPE tissue or the tissue surrogate mixture, the lysozyme tissue surrogates were heated at 100°C for 2 h and the 50 mM Tris-HCl, pH 4, 2% SDS extraction buffer was supplemented with 0.2 M glycine as an additional formaldehyde scavenger. The average particle size of samples extracted at atmospheric pressure was 200±55 nm, suggesting that the solubilized fraction remained highly cross-linked, which was confirmed by SDS-PAGE (Figure 6). There was a rapid decrease in particle size with increasing hydrostatic pressure, with a measured average of 40–50 nm after 10,000 psi (Figure 5). Recovery of monomeric protein, as shown by SDS-PAGE, indicated that the decrease in particle size corresponded to the reversal of formaldehyde-induced protein cross-links. These results suggest that elevated hydrostatic pressures improves the recovery of proteins from FFPE tissue surrogates by hydrating and promoting solubilization of the protein aggregates, allowing for the subsequent reversal (by hydrolysis) of formaldehyde-induced protein adducts and cross-links.

Most reported methods for proteomic analysis of FFPE tissue [13]–[16], [18] require both heat treatment and tryptic digestion in order to yield an extract that can be analyzed by gel electrophoresis or LC/MS. The use of elevated hydrostatic pressure, however, allows these two steps to be decoupled, facilitating the recovery of intact proteins devoid of formaldehyde adducts or cross-links following heat treatment under pressure. This outcome can be seen in the SDS-PAGE gel profiles of Figure 1 and Figure 3. This capability suggests that intact proteins recovered from FFPE tissue may be suitable for proteomic studies involving protein or antibody arrays [42], [43].

In summary, we have used SDS-PAGE, and LC/MS to investigate the recovery of proteins from a multi-protein tissue surrogate, composed of 5 proteins of differing abundance, molecular weight, structural class, and pI, subjected to heat treatment augmented by elevated hydrostatic pressure. Our results demonstrate that treatment of the tissue surrogates at 80–100°C under elevated pressure yields quantitative solubilization of protein. Our results also indicate that the addition of elevated hydrostatic pressure dramatically improves the LC/MS analysis of the FFPE tissue surrogate, with each protein identified with comparable sequence coverage to its counterpart in the native, unfixed protein mixture. Analysis of the LC/MS/MS data also suggests that elevated pressure aids in the reversal of formaldehyde-induced protein adducts and cross-links. LC/MS/MS of surrogates extracted with heat and elevated pressure identified a number of full-length tryptic peptides with false identification rates comparable to that of the unfixed protein mixture. Tissue surrogates extracted with heat alone had relatively fewer peptides identified (7.8–16% that of the unfixed protein mixture), and a false ID rate of 42% (pH 4) and 100% (pH 8). The high-pressure assisted extraction method also improved protein recovery from FFPE mouse liver over heat extraction alone. Our mechanistic studies suggest that the partial inhibition of the cross-link reversal reaction by elevated pressure is more than offset by the ability of elevated hydrostatic pressure to hydrate the inner core of the proteins, induce protein unfolding, and reduce the size of the protein aggregates, thus allowing full access of the formaldehyde adducts and cross-links to the reversal buffer. Accordingly, these experiments further establish that elevated hydrostatic pressure treatment is a promising approach for improving the recovery of proteins from FFPE tissues for proteomic analysis.

## Supporting Information

Data S1 List of Peptides identified by LC/MS/MS for each multi-protein surrogate. The single data file includes the peptide lists for native, unfixed protein, FFPE surrogates extracted at pH 4 with and without elevated pressure and FFPE surrogates extracted at pH 8 with and without elevated pressure.
(0.09 MB XLS)
